# Evaluating the eutrophication risk of artificial lagoons–case study El Gouna, Egypt

**DOI:** 10.1007/s10661-022-10767-5

**Published:** 2022-12-03

**Authors:** Omnia Abouelsaad, Elena Matta, Reinhard Hinkelmann

**Affiliations:** 1 Chair of Water Resources Management and Modeling of Hydrosystems, Technische Universität Berlin, Gustav-Meyer-Allee 25, 13355 Berlin, Germany; 2 Irrigation and Hydraulics Department, Mansoura University, Mansoura City, Egypt; 3 Politecnico Di Milano - Department of Electronics, Information, and Bioengineering, Environmental Intelligence for Global Change Lab, Milano, Italy

**Keywords:** TELEMAC, EUTRO-WAQTEL, Water quality, Dissolved oxygen, Phytoplankton biomass, Manmade coastal lagoons, Eutrophication

## Abstract

Eutrophication problem in El Gouna shallow artificial coastal lagoons in Egypt was investigated using 2D TELEMAC-EUTRO-WAQTEL module. Eight reactive components were presented, among them dissolved oxygen (DO), phosphorus, nitrogen, and phytoplankton biomass (PHY). The effect of warmer surface water on the eutrophication problem was investigated. Also, the spatial and temporal variability of the eutrophication was analyzed considering different weather conditions: tide wave, different wind speeds and directions. Moreover, effect of pollution from a nearby desalination plant was discussed considering different pollution degrees of brine discharge, different discharge quantities and different weather conditions. Finally, new precautions for better water quality were discussed. The results show that tide wave created fluctuations in DO concentrations, while other water quality components were not highly influenced by tide’s fluctuations. Also, it was found that high water temperatures and low wind speeds highly decreased water quality producing low DO concentrations and high nutrients rates. High water quality was produced beside inflow boundaries when compared to outflow boundaries in case of mean wind. Moreover, the results show that the average water quality was not highly deteriorated by the nearby desalination operation, while the area just beside the desalination inflow showed relatively strong effects. Different weather conditions controlled the brine’s propagation inside the lagoons. Moreover, increasing the width of the inflow boundaries and injecting tracer during tide and mean wind condition are new precautions which may help to preserve the water quality in a future warmer world. This study is one of the first simulations for eutrophication in manmade lagoons.

## Introduction

The water eutrophication problem is considered one of the most challenging environmental issues resulting in destructive effects on aquatic ecosystems (Pal, 2020). It means the enrichment of the water bodies with excess components of nitrogen (N) and phosphorous (P), which cause oxygen depletion problems (Yang et al., 2008). Eutrophication leads to the degradation of cultural and social values of polluted water bodies. For instance, large hypoxic or dead zones are formed in high polluted ecosystems, which result in the reduction of fish and shellfish production (Wurtsbaugh, 2019). Also, coral reefs are highly stressed and decline affected by eutrophication (Kroon et al., 2016). Up to 2012, the Fifth Global Environment Outlook (GEO-5) stated that more than 40% of the total world water bodies suffer from eutrophication. This environmental crisis has become a vital social issue involving a wide variety of stakeholders aiming at eutrophication reduction (Smetacek & Zingone, 2013). In North America and Europe, there are great successes in reducing the problem; however, many developing countries still suffer from lots of water eutrophication problems (Wurtsbaugh, 2019).

Nowadays, eutrophication problems are being increased affected by both anthropogenic activities and natural phenomena. For instance, the increasing anthropogenic activities such as agricultural, recreational and industrial interests, fossil fuel combustion, and many other economic growth activities are dramatically increasing aquatic nitrogen and phosphorus pollution (Wurtsbaugh, 2019). On the other hand, most water bodies are naturally negatively affected by a gradual slow process of nutrient enrichment as they age affected by sediments and algal decomposition. The geomorphology, mean water depth, wind, and tide are considered major natural hydrodynamic factors affecting eutrophication (de Jonge et al., 2002). Moreover, the expected natural climate change in the upcoming decades will also have direct and indirect effects on water eutrophication considering changes in metrological factors such as water temperature, precipitation patterns, solar radiation, and wind effects (Woznicki et al., 2016; Xia et al., 2016). For instance, the water temperature is considered a major environmental factor affecting water quality parameters such as algal bloom stimulation and increase of nutrients (Nazari-Sharabian et al., 2018). In a globally warming climate, air temperature is expected to increase by 1.1–6.4 °C by 2100, which, in turn, rapidly increases water temperature (Anthony et al., 2009). Also, any decrease in wind speeds can increase algae production and hinder the release of nutrients from sediments (George et al., 2007). In the expected climate change, the wind speeds are tending to decrease (with a negative effect on water quality) or increase (with a positive effect on water quality) affected by global warming depending on the studied location (Eichelberger et al., 2011).

In the last decades, researchers began to focus on the effect of the upcoming climate change and natural phenomena on the water quality and the eutrophication problem using field investigations or numerical models, which are widely accepted in complex domains such as coastal areas. For instance, Kim et al., (2018), Liu et al. (2019), Sepulveda-Jauregui et al. (2018), and Trombetta et al. (2019) investigated the effect of increasing water temperature on different water quality parameters. They found out that increasing water temperature played a major role in reducing water quality in water systems. Also Deng et al. (2018), Kunarso et al. (2018), Marlina and Melyta (2019), Morgan et al. (2019), Wirasatriya et al. (2018), and Zhang et al. (2017) studied the effect of different wind speeds on water eutrophication problems. They found out that decreasing wind speed can increase water pollution, e.g., the decrease of dissolved oxygen (DO) concentrations and the increase of water pollutants of phosphorus and nitrogen. Moreover, Fatema et al. (2016), Gasim et al. (2015), and Purnaini et al. (2018) investigated the high effect of tidal waves as a main meteorological parameter on water quality in different water systems.

In particular, eutrophication in coastal water areas is a common phenomenon, which needs more special awareness for many reasons. For instance, coastal areas are destinations for approximately 45% of the human population, which placed these systems under severe anthropogenic stress affected by the increasing nutrient enrichment from unprecedented agricultural, industrial, and urban growth (Kay & Alder, 2005; Wurtsbaugh et al., 2019). Also, coastal areas are more vulnerable to global warming than open estuaries, in which water temperature increases more rapidly reaching approximately four times greater than that of the ocean (Anthony et al., 2009; Nixon et al., 2004). Moreover, coastal areas have complex physical processes with the complexity of describing eutrophication processes affected by their transitional locations (Fernández et al., 2012). Consequently, efficient and effective management of water eutrophication in coastal areas is a challenge considering their societal importance, increasing number of polluted coasts, high population and pollution, high effect of global warming and their complex interactions. Nevertheless, the issue of eutrophication problem in coastal areas, estuaries, and lagoons has attracted less attention from the scholarly community, when compared to lakes investigations (Wurtsbaugh, 2019). More particular, few articles were published considering water pollution in lagoons compared to other coastal areas and estuaries (Ménesguen & Lacroix, 2018). Moreover, to the authors’ knowledge, no previous studies investigated the eutrophication problem in manmade lagoons.

In this work, a two-dimensional numerical model using the EUTRO-WAQTEL module of the TELEMAC-2D modeling system was applied for artificial coastal lagoons in El Gouna City located in Egypt. El Gouna’s artificial lagoons are connected to the Red Sea and present a touristic and economic environment in Egypt. The lagoons are getting stressed day by day affected by touristic wastes, sewage outflow from lots of hotels, boat leakage, and the outflow from an adjacent desalination plant. Investigation of the hydrodynamics and water quality of El Gouna artificial lagoons has attracted consideration since 2018. Al-Jabari (2018) carried out a field investigation for bathymetry and tide wave affecting the lagoons (Al-Jabari, 2018). Moreover, Abouelsaad et al. (2022a) studied the hydrodynamics of El Gouna lagoons under different weather conditions and tracer transport scenarios. They found that the hydrodynamics of the lagoons were highly affected by the surrounding weather conditions of tide and wind. Also, Abouelsaad et al. (2022b) applied the simple water quality module of TELEMAC-O_2_-WAQTEL for the simulation of DO concentration under different temperatures and weather conditions. They also discussed the negative effect of binary effluents from the nearby desalination plant on DO in the lagoons. O_2_-WAQTEL module is a simple approach, which investigates only DO, organic load (L), and ammoniacal load (NH_4_) without the full complexity of the manifold biological interactions and feedbacks. Herein, the authors tended to apply the more complicated and more advanced EUTRO-WAQTEL module. The EUTRO-WAQTEL module can be applied to investigate the oxygenation of any water body and is not restricted to modeling reaeration and the global oxidizable load.

The advanced eutrophication module of EUTRO-WAQTEL was used to model phytoplankton biomass (PHY) represented by ChlA, DO, nitrogen (N), and phosphorous (P) in the lagoons. To the authors’ knowledge, this study is the first published application of the EUTRO-WAQTEL-TELEMAC module for simulating the eutrophication problem in water systems such as the lagoons in El Gouna. The model provides a spatial and temporal simulation of the eutrophication problem considering the domain hydrodynamics driven by tide, wind, and the lagoons’ connection with the Red Sea and the nearby desalination plant. The aim of this work is investigating the eutrophication problem in the lagoons. Firstly, a sensitivity analysis of nine input parameters in the EUTRO-WAQTEL model was carried out discussing their effect on water quality. Afterwards, the effect of different weather conditions on the physical, chemical, and biological characteristics of water was analyzed such as the tide wave, the wind speed, and the wind direction. Also, the expected climate changes of the predicted high temperatures and low wind speeds on water quality were investigated owing to the definite tendency toward lowering the strength of wind speed in the Red Sea.

On the other hand, this study sheds new light on the effect of desalinated water on the eutrophication problem in the receiving coastal lagoons. In this regard, the negative effect of pollution from the nearby desalination plant on the eutrophication process in the lagoons was investigated considering different weather conditions and different pollution intensity of desalination’s effluent. Moreover, new precautions or changes in the hydraulics and the operation system of the nearby desalination plant have been discussed for the preservation of the desired water quality in the studied lagoons aiming for better dealing with the surrounding industrial and economical revolution and the expected climate change in the future.

## Study area

**Fig. 1:**
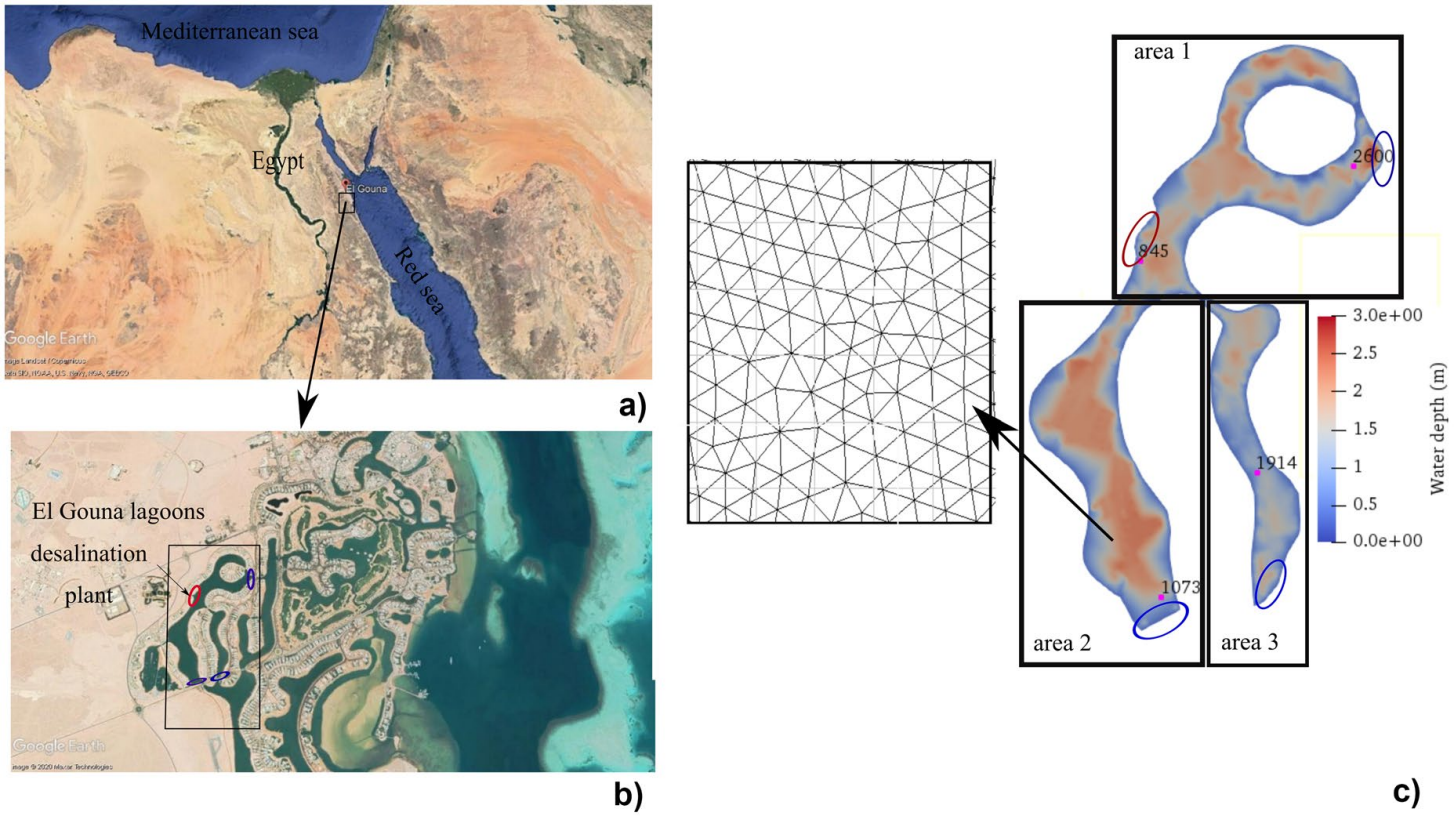
Location of the study area in Egypt: **a**) El Gouna City, **b**) capture of El Gouna lagoons (adapted by the author from Google Earth (2020), and **c**) computational domain showing observation points, water depth (m), inflow and outflow boundaries (blue: open boundaries to neighboring lagoons, red: open boundary to desalination plant), subdivision of the domain for three areas (area 1 and 2 and 3) for comparing targets and zoom of the unstructured triangular mesh

El Gouna city is located 20 km north of Hurghada City, the capital of the Red Sea governorate in Egypt (see Fig. 1a). It is considered one of the most significant tourist attraction sites in Egypt enriched by tourism activities. El Gouna has a system of artificial lagoons, which were constructed for touristic purposes. The studied domain consists of two connected artificial lagoons with a perimeter of approximately 5000 m and a surface area of 230,000 m^2^ with a mean water depth of not more than 2.7 m (see Fig. 1c). Those lagoons are highly stressed by the sewage outflow of touristic hotels, domestic wastes, and boat leakage (Abouelsaad et al., 2022a). The lagoons exchange their water with the Red Sea through three narrow water inlets. Moreover, as the whole city strongly depends on the desalination of salted water through three desalination plants, one nearby desalination plant throws its effluents into the lagoons and is considered the fourth temporary water inlet. Figure 1c shows the four mentioned boundaries with the Red Sea and the desalination plant. The blue ovals represent Dirichlet boundary conditions for water levels of boundaries connected to the Red Sea and the red oval represents the tracer’s discharge and concentration from the nearby desalination plant assigned also as Dirichlet boundary conditions.

In 2018, Al-Jabari (2018) draws a picture of the importance of the studied artificial lagoons in El Gouna by studying their bathymetry and tide data. He tracked the water depths for approximately 1700 locations in the whole lagoons. Water depths were not more than 2.7 m. Also, he simulated the tide force affecting the lagoons, which was simulated as a sinusoidal wave, in which every tide wave had an amplitude of 0.6 m every half-day. Since 2013, records of wind speeds and directions have been recorded by the metrological station beside the Technische Universität Berlin Campus El Gouna every 10 min for the whole day. The weighted monthly average wind speed and direction for two years from 2015 to 2017 were calculated to range from 4 to 8.24 m/s with an average value of 5.84 m/s from the northwest direction. Also, the maximum wind speed was found to equal 20.8 m/s blowing from the southeast (Abouelsaad et al., 2022a).

## TELEMAC EUTRO-WAQTEL model

For a proper water quality simulation, the water depths and vertically averaged velocity variations must be accurately investigated. In this regard, TELEMAC-2D numerical module was firstly applied to analyze the shallow water flow in the studied lagoons. The shallow water governing equations are the continuity equation, the momentum equations in x- and y-directions and finally the tracer equation (Hervouet & Ata, 2017). Also, Flather’s approach (Flather, 1976) was considered to simulate the wind shear stress. TELEMAC-2D was then coupled with EUTRO-WAQTEL module for investigations of water quality.

EUTRO-WAQTEL module gives a comprehensive insight into the oxygenation of water by computing the oxygen reaeration and the overall oxidizable load. Moreover, it takes into account the effect of planktonic plant photosynthesis and models the nitrogenous and phosphorus nutrients and their effect on phytoplankton. EUTRO module requires setting the values of more than 25 parameters to simulate eight tracers: PHY defined as the equivalent ChlA concentration, DO, dissolved mineral phosphorus assimilable by phytoplankton (PO_4_), degradable phosphorus not assimilable by phytoplankton (POR), dissolved mineral nitrogen assimilable by phytoplankton (NO_3_), degradable nitrogen that cannot be assimilated by phytoplankton (NOR), ammoniacal load assimilable by phytoplankton (NH_4_), and organic load (L) (Hervouet & Ata, 2017). The tracer conservation equation is given as follows (Hervouet & Ata, 2017):

1 $$\frac{\partial {C}_{i}}{\partial t}+u\frac{\partial {C}_{i}}{\partial x}+v\frac{\partial {C}_{i}}{\partial y}-\frac{\partial }{\partial x}({\nu }_{t,t}\frac{\partial {C}_{i}}{\partial x})-\frac{\partial }{\partial y}({\nu }_{t,t}\frac{\partial {C}_{i}}{\partial y})={F}_{i}$$

where *c* is the tracer concentration (mg/l or μg/ l), *u* and *v* are the velocity components in x- and y-directions, respectively (m/s), $v_{t,t}$  is the turbulent diffusivity (m^2^/s), $F_i$ is a source or sink accounting for water quality interactions of the respective tracer *i* (mg/l/s).

The eight variables are combined in four interacting systems introduced in the following phytoplankton kinetics, nitrogen cycle, phosphorus cycle, and DO balance.

### Phytoplankton kinetics

Phytoplankton mainly consists of unicellular algae that live in water and play a basic role in the food chain. It absorbs mineral elements such as nitrogen, phosphorus, and iron and transforms these materials into organic matter using the light energy received by the chlorophyll they contain via the photosynthesis process (Chao et al., 2010). The governing equation for phytoplankton considering its production and losses is:

2 $${F}_{PHY}=\left(CP-DP\right)*[PHY]$$

where *F*_*PHY*_ is the phytoplanktonic biomass, *CP* and *DP* are algal growth and disappearance rates in (1/day).

The growth rate of a phytoplankton population is a physiological process often limited by temperature, nutrients and light, as shown in Eqs. (3) and (4) (Guildford & Hecky, 2000; Örnólfsdóttir et al., 2004; Wu et al., 2009):

3 $$CP= {C}_{\mathrm{max}}*{g}_{1}*\mu \left(\mathrm{lim}\left(\mathrm{phosphate },\mathrm{ nitrate}\right)\right)*{K}_{r1}*{\alpha }_{1}$$

4 $$\begin{aligned}&\mu \left(\mathrm{lim}\left(\mathrm{phosphate},\mathrm{ nitrate}\right)\right)\\& \quad=\mathrm{min}\ (\frac{{[PO}_{4}]}{KP+{[PO}_{4}]},\frac{{[NO}_{3}]+{[NH}_{4}]}{KN+{[NO}_{3}]+{[NH}_{4}]}\end{aligned}$$

where *C*_max_ is the maximum growth rate (1/day), *g*_*1*_ is the temperature effect on algal growth (-), *μ* (lim (phosphate, nitrate)) is the growth limited by the availability of either phosphate, *PO*_*4*_, or the sum of nitrate, *NO*_*3*_, and ammonium, *NH*_*4*_ (-), *K*_*r1*_ represents the effect of sunlight on algal growth (-), ${\alpha }_{1}$ is the water toxicity coefficient for algae which equals to 1 in the presence of toxicity (-), *KP* is the half-saturation constant of phosphate (mgP/l), and *KN* is the half-saturation constant of nitrate (mgN/l).

In the EUTRO-WAQTEL module, the light limitation may be simulated by a depth-integrated Smith function, which does not represent photoinhibition. Herein, the light limitation was neglected to focus on the phytoplankton dynamics under nutrients.

On the other hand, PHY reduction rate is mainly governed by respiration, natural nonpredatory mortality and zooplankton grazing. In the WAQTEL module, the zooplankton grazing is not included as a separate state variable. Nonpredatory mortality accounts for all algal losses which are not explicitly accounted for such as the grazing term or other loss processes. The following equations describe the phytoplankton death rate (DP) as described in TELEMAC:

5 $$DP=\left(RP+MP\right)\ast g_2\ast\alpha_2$$

6 $$MP={M}_{1}+{M}_{2}*\left[PHY\right]$$

where *RP* is the phytoplankton respiration rate (1/day), *MP* is the algal disappearance rate due to nonpredatory mortality (1/day), *M*_1_ and *M*_*2*_ are nonpredatory mortality coefficients, *g*_*2*_ is the temperature effect on phytoplankton disappearance *(*= ${1.050}^{T-20}$), ${\alpha }_{2}$ is the water toxicity coefficient for algae (-), *T* is the temperature (°C).

### Phosphorus cycle

Phosphorus is considered a persistent and serious ecological problem in the eutrophication process through organic and inorganic phosphorus particles (Liu et al., 2006). Firstly, inorganic phosphorus is incorporated into the biomass during the phytoplankton growth process, while nonliving organic and inorganic particles are recycled during phytoplankton death and undergo mineralization or bacterial decomposition into inorganic phosphorus before utilization by phytoplankton (Wang et al., 2012). Particulate organic phosphorus settles into the sediment. Organic and inorganic phosphorus are modeled in EUTRO module as follows:

7 $${F}_{{PO}_{4}}= {(f}_{p}\left({(d}_{tp}*DP)-CP)*\left[PHY\right]\right)+{K}_{320}*{g}_{2}*\left[POR\right]$$

8 $$\begin{aligned}{F}_{POR}=&\ \left({f}_{p}*\left(1-{d}_{tp}\right)*DP*\left[PHY\right]\right)\\&-{K}_{320}*{g}_{2}*\left[POR\right]-\frac{{f}_{POR}}{h}\end{aligned}$$

where ${f}_{p}$ is the phosphorus content per *PHY* (mgP/mgChlA), ${d}_{tp}$ is the phosphorus percentage that is assimilable in dead phytoplankton (%), *K*_*320*_ is the mineralization rate from *POR* to *PO*_*4*_ at 20 °C (1/day), ${f}_{POR}$ is non-algal organic phosphorus deposition flux (mg/m^2^/s), which equals the rate of sedimentation of non-algal organic phosphorus (*W*_*POR*_), *h* is the water depth (m).

### Nitrogen cycle

During the nitrogen cycle, three nitrogen variables are considered (Eqs. 9, 10, and 11): NO_3_, NOR, and NH_4_. Firstly, inorganic nitrogen is being incorporated into the biomass during phytoplankton growth process, while nonliving organic and inorganic particles are recycled during the phytoplankton death. Nonliving organic nitrogen undergoes mineralization or bacterial decomposition into inorganic nitrogen before utilization by phytoplankton (Wang et al., 2012). Particulate organic nitrogen settles into the sediment.

9 $${F}_{{NO}_{3}}= {(f}_{n}\left({(d}_{tn}*DP)-CP)*\left[PHY\right]\right)+{K}_{520}*{g}_{2}*\left[{NH}_{4}\right]$$

10 $$\begin{aligned}{F}_{NOR}=&\left({f}_{n}*\left(1-{d}_{tn}\right)*DP*\left[PHY\right]\right)\\&-{K}_{620}*{g}_{2}*\left[NOR\right]-\frac{{f}_{NOR}}{h}\end{aligned}$$

11 $$\begin{aligned}{F}_{{NH}_{4}}=& \ ({f}_{n}*{(d}_{tn}*DP-{R}_{n}*CP))*\left[PHY\right]\\&+{K}_{620}*{g}_{2}*\left[NOR\right]-{K}_{520}*{g}_{2}*\left[{NH}_{4}\right]\end{aligned}$$

where ${f}_{n}$ is the nitrogen content per *PHY* (mgN/mgChlA), *K*_*520*_ is the nitrification rate at 20 °C (1/day), ${d}_{tn}$ is the nitrogen percentage that is assimilable in dead phytoplankton (%), *K*_*620*_ is the mineralization rate from *NOR* to NO_3_ at 20 °C (1/day), ${f}_{NOR}$ is non-algal organic nitrogen deposition flux (mg/m^2^s), which equals the rate of sedimentation of non-algal organic nitrogen (*W*_*NOR*_), and *R*_*n*_ is the nitrogen proportion that is assimilated from ammonium (-).

### Dissolved oxygen balance

#### Organic load

12 $$\begin{aligned}{F}_{L}=&\ f*\left({M}_{1}+{M}_{2}*\left[PHY\right]\right)*\left[PHY\right]\\&-{K}_{120}*{g}_{3}*\left[L\right]-\frac{{f}_{LOR}}{h}\end{aligned}$$

where *f* is the amount of oxygen produced by photosynthesis (mgO_2_/l), *K*_*120*_ is the degradation rate of *L* at 20 °C (1/day), *g*_*3*_ is the temperature effect on degradation of *L* (-), ${f}_{LOR}$ is the non-algal *L* deposition flux (mg/m^2^s) which is equal to ${W}_{LOR}$, and ${W}_{LOR}$ is the rate of sedimentation of non-algal L (m/s).

#### Dissolved oxygen

13 $$\begin{aligned}{F}_{{O}_{2}}=& \ \left(f*\left(CP-RP\right)*\left[PHY\right]\right)-n{K}_{520}*{g}_{2}*\left[{NH}_{4}\right]\\&-{K}_{120}*{g}_{3}*\left[L\right]+{K}_{2}*{g}_{4}*({C}_{S}-{[O}_{2}])-\frac{BEN}{h}\end{aligned}$$

where *n* is the quantity of oxygen consumed by nitrification (mgO_2_/mgNH_4_), ${K}_{2}$ is the gas-exchange coefficient between water and atmosphere at 20 °C (1/day), *g*_*4*_ is the temperature effect on reaeration (-), *C*_*s*_ is the oxygen saturation concentration (mg/l), and *BEN* is the benthic oxygen demand (mgO_2_/m^2^/day).

Reaeration, as one of the most important parameters affecting DO, is affected by water depth, velocity, the slope of the stream, diffusion coefficient, kinematic viscosity, Froude number, or Reynolds number and the head loss (Haider et al., 2013). In the TELEMAC WAQTEL module, four formulas may be considered in reaeration coefficient calculations at 20 °C considering water depth and velocity: formula of Authority, formula of Owens et al. (De la Presa Owens & Innis, 1999), formula of Churchill et al. (Churchill et al., 1964), and formula of O’Connor and Dobbins (O’Connor & Dobbins, 1958). Also, there is another formula that combines three formulae considering ranges for water depth and flow velocity of each point of the mesh using TELEMAC WAQTEL indicated in TELEMAC manual. For all discussed *K*_2_ formulas, the value of *K*_2_ is inversely proportional to water depth and directly proportional to flow velocity (Abouelsaad et al., 2022b). For the aforementioned formulas, the computed reaeration coefficient is determined at 20 $^\circ \mathrm{C}$. Correction of the temperature variation is considered in the TELEMAC model.

The oxygen saturation density of water (*C*_s_) is mainly dependent on the water temperature. TELEMAC WAQTEL offers the two most common formulas to calculate *C*_s_: formula of Elmore and Hayes and formula of Montgomery (Richardson et al., 2001).

Also, TELEMAC-2D suggests values of benthic demand ranging from 0.007 to 7 gO_2_/m^2^/day at 20 °C depending on the type of soil and bacteria. Moreover, correlation of the effect of temperature variation on the benthic demand is considered in the simulation using TELEMAC model.

## Model parameters and simulation scenarios

### Model parameters

A two-dimensional model for simulating El Gouna lagoons was set up using TELEMAC-2D coupled with EUTRO-WAQTEL module, using an unstructured triangular grid created by Janet mesh generation’s program as a part of smile software. A suitable unstructured fine mesh was generated counting in a total of about 2700 nodes and 4800 triangular elements with a mesh resolution, which ensured grid convergence, i.e., changes in model results using a refined grid can be neglected. In TELEMAC-2D simulations, the method of characteristics was applied to solve the shallow water equations by applying linear discretization for the flow velocities and tracer, while the PSI method was used for calculations of water depth. Also, Strickler’s law with a magnitude of 40 m^1/3^/s was adopted for the calculation of bottom friction (Abouelsaad et al., 2022a). Finally, a constant initial condition for water elevation of 12 m above the mean sea level and initial zero velocities were initially applied over the entire domain. The prescribed values for cross-section characteristics and numerical schemes were obtained from a previous study carried out by Abouelsaad et al. (2022a).

TELEMAC-2D is then compiled with the EUTRO-WAQTEL module using a separate steering file, which includes the different physical and biochemical processes of the eutrophication problem as illustrated in Eqs. (2) to (13). The EUTRO module is applied for simulating the eight beforementioned tracers using approximately 25 parameters shown in Table 1. The table indicates the default values of the parameters suggested by the user’s manual of TELEMAC. The ranges of some parameters, which were previously discussed by literature research, are also indicated.

**Table 1:** Typical kinetic parameters in eutrophication model, their default values in TELEMAC EUTRO module and previous literature ranges

Parameter	Symbol	Default value	Ranges from literature
Maximum growth rate	*C*_max_	2.0/day	0.2–3.6 (Rigosi et al., 2011)
Temperature effect on algal growth	*g*_1_	T/20	
Half-saturation constant of phosphate	KP	0.005 mgP/l	0.0005–0.01 (Wool et al., 2006) 0.000013–0.0501 (Shimoda & Arhonditsis, 2016) 0.0005–0.08
Half-saturation constant of nitrate	KN	0.03 mgN/l	0.005–0.04 (Wool et al., 2006) 0.0021–0.0321 (Shimoda & Arhonditsis, 2016)
Algal toxicity coefficients	${\alpha }_{1}$	1; 0	
Phytoplankton respiration rate	RP	0.05/d	0.01–0.2 (Wool et al., 2006) 0.055–0.17 (Park, 1980)
Mortality coefficients	*M*_1_, *M*_2_	0.1; 0003	
Phosphorus content per PHY	*f*_*p*_	0.0025 mgP/mgChlA	
Phosphorus percentage assimilable in dead phytoplankton	*d*_*tp*_	0.5%	
Mineralization rate from POR to PO_4_ at 20 °C	*K*_320_	0.03/day	0.001–0.05 (Wool et al., 2006)
Organic phosphorus sedimentation rate	*W*_POR_	0.0 m/s	
Nitrogen content per PHY	*f*_*n*_	0.0035 mgN/mgChlA	
Nitrification rate at 20 °C	*K*_520_	0.35/day	0.025–0.60 (Park et al., 2013)
Rate of sedimentation of non-algal organic nitrogen	*W*_NOR_	0.0 m/s	
Nitrogen percentage assimilable in dead phytoplankton	*d*_*tn*_	0.5%	
Mineralization rate from NOR to NO_3_ at 20 °C	*K*_620_	0.0/d	0.001–0.075 (Wool et al., 2006)
Temperature effect on degradation of L	${g}_{3}$	1.047 T^−20^	1.02–1.09 (Park, 1980)
Rate of sedimentation of L	*W*_lOR_	0.0 m/s	
Degradation rate of L at 20 °C	*K*_120_	0.35/d	0.16–0.21 (Wool et al., 2006) 0.1–1.5 (Park, 1980)
Amount of oxygen produced by photosynthesis (mgo_2_/l)	*f*	0.15 mgO_2_/l	
Benthic demand	BEN	0.1 gO_2_/m^2^	0.007–7 (Tchobanoglous & Schroeder, 1985)
Effect of temperature on natural reaeration	*g*_4_	1.025 T^−20^	
Quantity of oxygen consumed by nitrification	*n*	5.2 mgO_2_/mgNH_4_	

Field data about water quality in the studied lagoons are not available. Consequently, the initial background values of the tracers were considered from a previous nearby field study in 2016 (Fahmy et al., 2016) who collected a total of 108 seasonal coastal water samples during the period from 2011 to 2013 to investigate the hydrography, heavy metals, nutrient salts, and petroleum hydrocarbons in the Red Sea. One of their studied regions is relatively near to our lagoons. Table 2 illustrates the initial simulated values of tracers as discussed in the mentioned study.

**Table 2:** Initial tracer values used in the simulations

PHY (μg/l)	PO_4_ (μg/l)	POR (μg/l)	NO_3_ (μg/l)	NOR (μg/l)	NH_4_ (μg/l)	L (mg/l)	DO (mg/l)
0.50	3	3	14.00	4.00	10	3.0	8.0

### Simulation scenarios

A sensitivity analysis of the eutrophication problem in the lagoons under the effect of nine different input parameters was firstly carried out using the EUTRO-WAQTEL module for simulations of water quality. The effect of every input parameter was discussed by computing the percental difference (%), which was derived as the difference between the results of water quality parameters corresponding to the maximum and minimum values of the investigated input parameter divided by the minimum simulated values. Although no data for model calibration were available, we could gain an insight into how the water quality is influenced by changes in chosen values and whether the results are in reasonable ranges. In all sensitivity analysis scenarios, the same weather condition of the tide (with an amplitude of 0.6 m two times daily) with the mean wind (5.84 m/s from the northwest direction) was adopted over a period of 6 days. Table 3 illustrates the investigated input parameters, their simulated values and the expected effect on water characteristics.

**Table 3:** Selected parameters for sensitivity analysis and their values

Parameter	Simulated values	Expected effect
Maximum growth rate (C_max_)	0.2–2.0–3.6/day	PHY—DO (direct relation) PO_4_ – NH_4_- NO_3_ (inverse relation) POR-NOR-L (indirect relation affected by PHY)
Half-saturation constant of phosphate (KP)	0.000013–0.005–0.0501 MgP/l	PHY—DO (direct relation) PO_4_ – NH_4_- NO_3_ (inverse relation) POR-NOR-L (indirect relation affected by PHY)
Half-saturation constant of nitrate (KN)	0.005–0.03–0.04 mgN/l	PHY -POR- DO (direct relation) PO_4_ – NH_4_- NO_3_ (inverse relation) POR-NOR-L (indirect relation affected by PHY)
Mineralization rate from NOR to NO_3_ at 20 °C (*K*_620_)	0.0–0.001–0.075/day	NH_4_ (direct relation) NOR (inverse relation) NO_3_ – DO (indirect relation affected by NH_4_)
Mineralization rate from POR to PO_4_ at 20 °C (*K*_320_)	0.001–0.03–0.05/day	PO_4_ (direct relation) POR (inverse relation)
Nitrification rate at 20 °C (*K*_520_)	0.025–0.35–0.60/day	NO_3_ (direct relation) NH_4_, DO (inverse relation) DO (inverse indirect relation)
Benthic demand (BEN)	0.1–0.8–1.5 gO_2_/m^2^	DO (inverse relation)
Degradation rate of L at 20 °C (*K*_120_)	0.1–0.35–1.5/day	L, DO (inverse relation)
Phytoplankton respiration rate (RP)	0.01–0.05–0.2/day	POR – NOR- PO_4_ –NH_4_- (direct relation) PHY- DO (inverse relation)

Further, different weather conditions were investigated considering different tide, wind, and temperature characteristics discussing their effects on the water quality of the studied artificial lagoons. For instance, the influence of three different water temperatures of 20 °C, 25 °C, and 30 °C and the effect of ups and downs resulting from the tide wave were simulated. Also, the effect of different wind speeds ranging from 8.24 to 4.01 m/s blowing from the mean wind direction of 322° (northwest direction) and the maximum blowing wind during the study period with a wind speed of 20.80 m/s from southeast direction was also investigated. Three observation points, shown in Fig. 1c, were chosen for comparing results in addition to the average values for the studied scenarios. The point’s numbers represent the point’s IDs. The points were chosen beside the three boundaries of the lagoons with the Red Sea, as shown in Fig. 1c.

Moreover, the effect of the main source of pollution from the nearby desalination plant was simulated. For the following scenarios, beside comparing the average values in the whole domain, point 845 just beside the desalination inflow (shown in Fig. 1c) was also investigated. Data about the water quality of brine discharge and its inflow rate was not available. Consequently, different percentages of variation in water quality (for instance 10%, 50% of the initial values in the lagoons, shown in Table 2) were assumed and discussed under the same brine discharge of 1 m^3^/s. For more clarification, 10% variation in water quality, for instance, means that the DO decreased by 10% from its initial value in the lagoons, while the nitrogen and phosphours increased by 10% from their initial values in the domain.

Also, the effect of different brine discharges with values ranging from 0.05 to 1 m^3^/s was investigated. Moreover, the effect of different weather conditions on the propagation of polluted water was conducted.

The influence of each studied scenario was investigated by calculating the relative percent difference (RPD), as shown in Eq. (14):

14 $$\%RPD\;=\;(difference\;between\;maximum\;and\;minimum\;values)/(average\;value)\;\;\;\;\;\;\;\;\;\;\;\;\;\;\;\;\;\;\;\;\;\;\;\;\;\;\;\;\;\;\;\;\;\;\;\;\;\;\;\;\;\;\;$$

Finally, precautions have been discussed for the preservation of the desired water quality in the studied lagoons aiming for better dealing with the surrounding industrial and economical revolution and the expected climate change in the future. In this regard, new scenarios have been simulated investigating the effect of changes in the hydraulics of the lagoons and different operation scenarios of the nearby desalination plant. For instance, the effect of increasing the real width of the three open boundaries with the Red Sea has been discussed. Moreover, the effect of increasing the water quality of the inflow water from the Red Sea by changing the input DO value as a main water quality indicator was analyzed. Also, the injection rapidity of the same quantity of brine disposals has been discussed (i.e., the comparison between rapid inflow of 0.4 m^3^/s in 1 day and slower inflow of 0.1 m^3^/s in 4 days and their effect on the water quality in the lagoons) in a trial to reduce their negative effects and help decision-makers in the city of El Gouna. Also, recommendations were given for decision-makers with regard to brine injection in specific weather conditions by simulating the effect of brine injection on water quality considering three different weather conditions: tide only, mean wind only, and tide with mean wind.

## Results and discussion

### Sensitivity analysis

#### Maximum growth rate and half-saturation constants

From Eqs. (2) to (4), the algal growth rate (CP) is mainly affected by the maximum phytoplankton growth rate (*C*_max_), algal sunlight (*K*_*r*1_), and the growth limited by the availability of either phosphate or the sum of nitrate and ammonium μ(lim(phosphate, nitrate)), which, in turn, is affected by either the half-saturation constant of phosphate or nitrate. The sensitivity of the lagoons was investigated considering different referenced values (indicated in Table 3) of the aforementioned parameters by computing percental difference (%) and neglecting the sun effect to concentrate on the effect of nitrogen and phosphours. Table 4 indicates the fluctuations in tracer values under different maximum growth rate values (*C*_max_), while Table 5 demonstrates the water quality variables under different half-saturation constant values of phosphate (KP).

**Table 4:** Effect of different maximum growth rates (C_max_) on the average values of the water quality variables in the whole domain

*C*_max_ (1/day)	PHY (μg/l)	PO_4_ (μg/l)	POR (μg/l)	NO_3_ (μg/l)	NOR (μg/l)	NH_4_ (μg/l)	L (mg/l)	DO (mg/l)
0.2	0.07	3.30	3.78	19.94	5.60	3.96	1.23	7.56
2	0.14	2.30	4.59	18.65	6.83	3.77	1.25	7.57
3.6	0.21	1.27	5.44	17.32	8.11	3.57	1.27	7.58
% Difference	200	− 61.52	43.92	− 13.14	44.82	− 9.85	3.25	0.26

**Table 5:** Effect of different half-saturation constant values of phosphate (KP) on the average values of the water quality variables in the whole domain

KP (mg/l)	PHY (μg/l)	PO_4_ (μg/l)	POR (μg/l)	NO_3_ (μg/l)	NOR (μg/l)	NH_4_ (μg/l)	L (mg/l)	DO (mg/l)
0.0501	0.07	3.26	3.81	19.89	5.65	3.95	1.23	7.56
0.005	0.14	2.30	4.59	18.65	6.83	3.77	1.25	7.57
0.000013	0.21	1.57	5.16	17.69	7.68	3.65	1.26	7.58
% Difference	200	− 51.84	35.43	− 11.26	35.93	− 7.59	2.44	0.26

Both the maximum phytoplankton growth rate and the phosphate half-saturation constant directly affect the algal growth rate, which in turn has a major effect on the overall water quality. For instance, as shown in Tables 4 and 5, the average PHY highly increased by 200% percental difference, while the average L and DO received minor changes with differences not more than 3.25%. Moreover, PO_4_, NH_4_, and NO_3_ decreased owing to the increasing algal growth with percental differences of 61.52%, 51.84% for PO_4_, 9.85%, 7.59% for NH_4_ and 13.14%, 11.26% for NO_3_ affected by changes in the maximum phytoplankton growth rate and the phosphate half-saturation constant, respectively. POR and NOR, on the other hand, increased by rates equal to 43.92%, 35.43% and 44.82%, 35.93%, respectively.

Besides, the domain was also investigated under different values of nitrate half-saturation constant (KN) since the growth rate was limited by the availability of either phosphate or the sum of nitrate and ammonium. The analysis of the results indicated that the domain was not sensitive to changes in the nitrogen half-saturation constant.

#### Mineralization rates

From Eqs. (10) and (11), the mineralization rate from NOR to NO_3_ at 20 °C (*K*_620_) directly affects NOR and NH_4_, which, in turn, affects the DO concentrations and the NO_3_ (Eqs. 9 and 13). Table 6 illustrates the sensitivity of the aforementioned water quality variables to different mineralization rates.

**Table 6:** Effect of different mineralization rates from NOR to NO_3_ (*K*_620_) on the average values of the water quality variables in the whole domain

*K*_620_ (1/day)	NO_3_ (μg/l)	NOR (μg/l)	NH_4_ (μg/l)	DO (μg/l)
0.0	18.65	6.83	3.77	7.57
0.001	18.66	6.81	3.78	7.57
0.075	19.27	5.51	4.47	7.57
% Difference	3.32	− 19.33	18.57	0.0

It can be observed from Table 6 that the variation in *K*_620_ values has relatively high effects on the average NOR and NH_4_ and has a minor impact on the average NO_3_ and no effect on DO. For instance, the average NH_4_ increased from 3.77 μg/l to reach a value of 4.47 μg/l with 18.57% increasing rate owing to the increase of *K*_620_ value from 0.0 to 0.075/day. Moreover, the average NOR decreased by approximately 20% percental difference. The indirect effect of the *K*_620_ increase had a slighter outcome on NO_3_, which increased by 3.32% by increasing *K*_620_ value. On the other hand, DO was not affected by changes in *K*_620_ values.

On the other hand, the mineralization rate from POR to PO_3_ at 20 °C (*K*_320_) affected only the values of PO_4_, POR (Eqs. 7 and 8) as indicated in Table 7. For instance, the average POR decreased by approximately 12% owing to the increase in *K*_320_ from 0.001 to 0.05, while the average PO_4_ increased from 1.95 to 2.51 μg/l owing to the same increase in *K*_320_.

**Table 7:** Effect of different phosphorus mineralization rates from POR to PO_3_ (*K*_320_) on the average values of the water quality variables in the whole domain

K_320_ (1/day)	PO_4_ (μg/l)	POR (μg/l)
0.001	1.95	4.96
0.03	2.30	4.59
0.05	2.51	4.37
% Difference	28.72	-11.90

#### Nitrification rate

From Eqs. (9) and (11), the nitrification rate at 20 °C (*K*_520_) directly affects the values of NO_3_ and NH_4_. DO concentration is indirectly influenced by the nitrification rate owing to its effect on NH_4_, as shown in Eq. (13). Table 8 illustrates the effect of different nitrification rate conditions on NO_3_, NH_4_, and DO in the lagoons.

**Table 8:** Effect of different nitrification rates on the average values of the water quality variables in the whole domain

*K*_520_ (1/day)	NO_3_ (μg/l)	NH_4_ (μg/l)	DO (mg/l)
0.025	13.78	8.64	7.58
0.35	18.65	3.77	7.57
0.6	19.99	2.43	7.57
% Difference	45.07	− 71.88	− 0.13

It can be observed from the indicated average values in Table 8 that NH_4_ and NO_3_ are highly sensitive to the variations of the nitrification rate (*K*_520_). For instance, NH_4_ reduced from 8.64 to 2.43 μg/l and NO_3_ increased by approximately 45% owing to the variation in *K*_520_. On the other hand, the DO was slightly affected by the aforementioned nitrification rate increase.

#### Benthic demand

As indicated in Eq. (13), the benthic demand only negatively affects the DO in the water domain. TELEMAC water quality manual suggests values of benthic demand ranging from 0.007 to 7 gO_2_/m^2^/day depending on the wastes coming to the water domain. As El Gouna City is not highly polluted, the lagoons were investigated under values of benthic demand that do not exceed 1.5 gO_2_/m^2^/day. The average DO values of 7.57, 6.82, and 6.07 mg/l corresponded to benthic demand values of 0.1, 0.8, and 1.5 gO_2_/m^2^/day. This reflects the high negative consequences of high benthic demand on the DO, which is considered the most vital parameter of aquatic life.

#### Degradation rate of L at 20 °C

As indicated in Eqs. 12 and 13, both L and DO are influenced by the degradation rate of L, which varies between 0.1 and 1.5/day. Table 9 illustrates the negative effect of the L degradation load on water quality variables. It can be observed that the degradation rate highly affected L as its average value dropped from 2.29 to 0.33 mg/l by more than 85% decrease percentage due to the increase of the degradation rate. Also, the DO decreased from 7.81 to 7.57 mg/l owing to the same variation of degradation rate.

**Table 9:** Effect of degradation load of L on the average values of the water quality variables in the whole domain

Degradation rate (1/day)	L (mg/l)	DO (mg/l)
0.1	2.29	7.81
0.35	1.25	7.57
1.5	0.33	7.47
% Difference	− 85.59	− 4.35

#### Phytoplankton respiration rate

Finally, the lagoons were investigated under different values of phytoplankton respiration rates considering values of 0.01,0.05, and 0.2/day. Table 10 illustrates the sensitivity of the water quality in the lagoons to the variation in respiration rate. It can be observed that the respiration rate has a small effect on approximately all water quality variable with differences not more than 2.2% for all variables except PHY and PO_4_ which were relatively highly affected by − 20% and 8.44% percental differences affected by increasing RP, respectively.

**Table 10:** Effect of respiration rate on the average values of the water quality variables in the whole domain

RP (1/day)	PHY (μg/l)	PO_4_ (μg/l)	POR (μg/l)	NO_3_ (μg/l)	NOR (μg/l)	NH_4_ (μg/l)	L (mg/l)	DO (mg/l)
0.01	0.15	2.25	4.62	18.59	6.82	3.76	1.25	7.57
0.05	0.14	2.30	4.59	18.65	6.82	3.77	1.25	7.57
0.2	0.12	2.44	4.52	18.83	6.72	3.79	1.24	7.57
% Difference	− 20.00	8.44	− 2.16	1.29	− 1.47	0.80	− 0.80	0.00

#### Sensitivity analysis conclusions

From the aforementioned sensitivity analysis of nine input water quality parameters, it can be concluded that the maximum growth rate (*C*_max_), the half-saturation constant of phosphate (KP) and the nitrification rate at 20 °C (*K*_520_) have the most obvious effect on most of the water quality variables. For instance, both the maximum growth rate (*C*_max_) and the phosphate half-saturation constant (KP) are highly affecting PHY, NOR, PO_4_, and POR, while the nitrification rate at 20 °C (*K*_520_) has obvious effect on both NO_3_ and NH_4_.

On the other hand, the degradation rate of L is highly affecting L, while DO is influenced mainly by the benthic demand. It can be concluded that the water quality in the lagoons may be highly sensitive to any change in input parameters.

Although we have no field measurements, we think we have reasonable values for the discussed parameters and gained an insight into the sensitivity of the lagoons for each simulated parameter. Therefore, the suggested reference values indicated in Table 1 were applied to conduct the following scenarios, in which the effect of different water temperatures, different weather conditions, and the effect of the pollution from the nearby desalination plant was conducted.

#### Temperature impacts

**Fig. 2:**
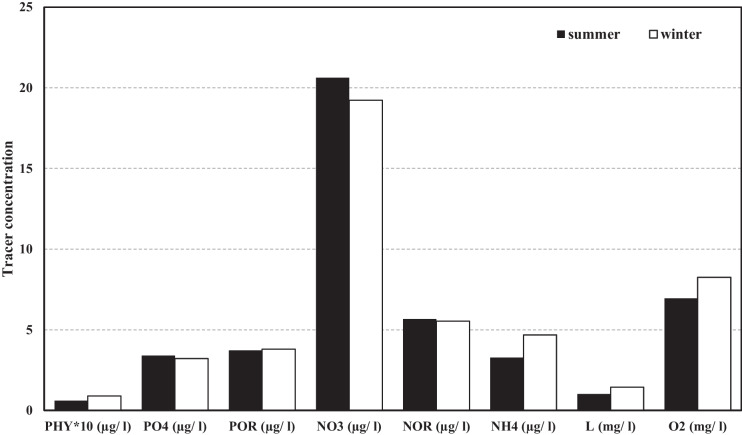
Seasonal variation of water quality variables during summer and winter

The average water temperature in El Gouna City is calculated to be approximately 25 °C with a minimum value of 20.6 °C in winter and a maximum value of 30 °C in summer (Abouelsaad et al., 2022a). Consequently, the domain water quality was investigated under the aforementioned water temperatures. Figure 2 illustrates the effect of different water temperatures on the water quality parameters and the eutrophication problem after 6 days of simulations.

It can be concluded from Fig. 2 that the changes in water temperature affects most of the water quality variables. For instance, the increase in water temperature decreased the average DO from 8.25 to 6.94 mg/l and PHY from 0.09 to 0.06 μg/l corresponding to percental decreases of approximately 16% and 33%, respectively. Also, NO_3_ and PO_4_ increased from 19.23 to 20.63 μg/l and 3.22 to 3.40 μg/l in summer, respectively, while high temperatures had smaller consequences on NOR and POR, with difference percentages of not more than 2.5%. On the other hand, the warmer water had positive effects on L and NH_4_, which decreased from 1.44 to 1.02 mg/l and 4.68 to 3.28 μg/l owing to the decrease in removal rates.

### Impacts of hydrodynamics

#### Tide wave

**Fig. 3:**
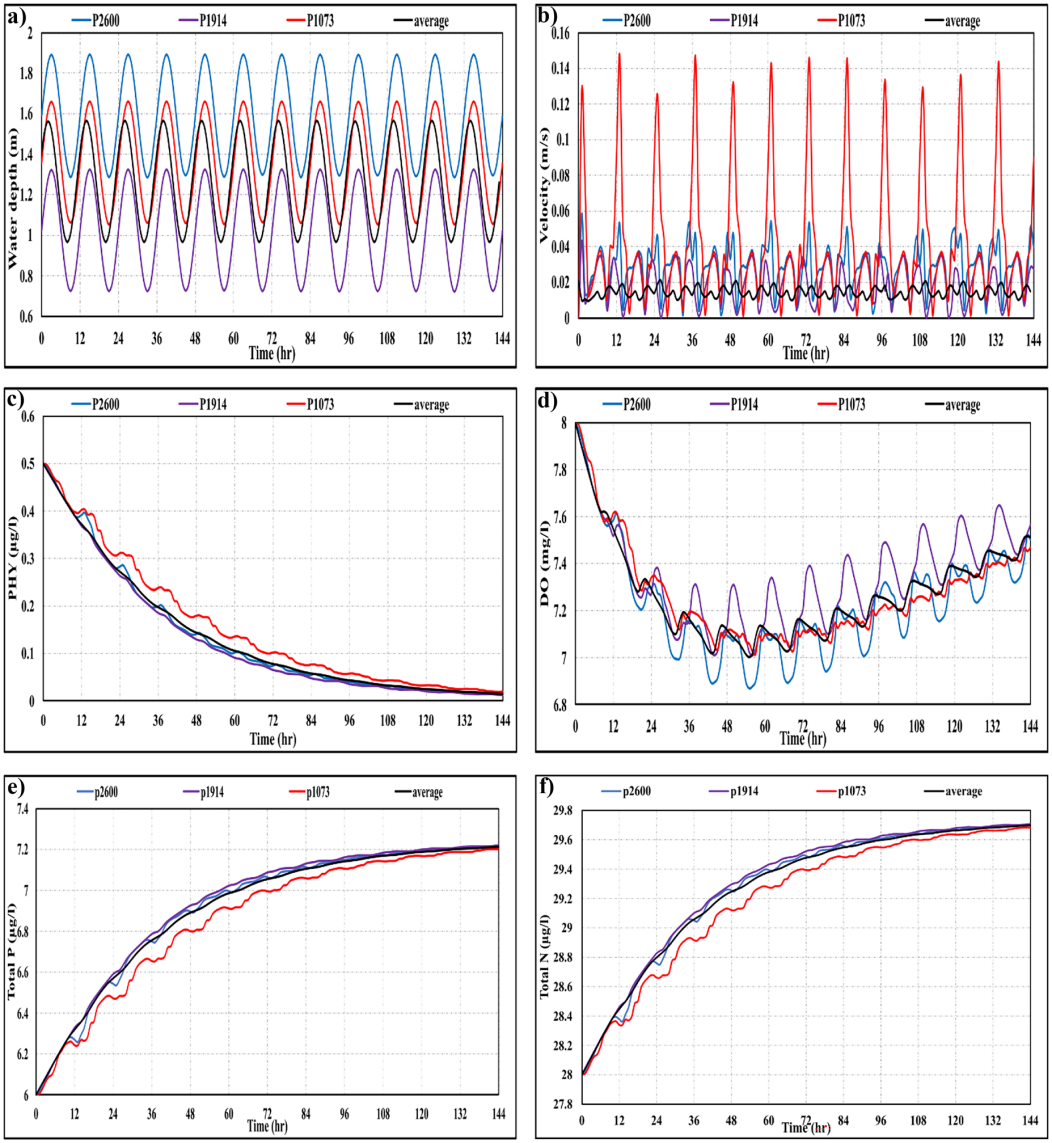
Effect of tide wave at three observation points and the average values on **a**) water depth (m), **b**) flow velocity (m/s), **c**) phytoplankton biomass (μg/l), **d**) DO (mg/l), **e**) total P (μg/l), and **f**) total N (μg/l)

Tide as a natural phenomenon can possibly affect water quality characteristics by moving pollutants upstream during high tide and downstream at low tide. In this regard, the effect of the tidal wave with 0.6 m amplitude on the eight water quality variables affecting the lagoons was studied. Figure 3 indicates the temporal fluctuation of these water quality variables (i.e., PHY, DO, total amount of phosphate (TP), which equals the summation of POR and PO_4_, and total amount of nitrate (TN), which equals the summation of NO_3_, NH_4_ and NOR). It indicates the difference in water quality at three observation points beside the three boundaries with the Red Sea, the domain-averaged values and the corresponding water depths and velocities. As shown in the figure, both the temporal average DO and the corresponding values of water depth and flow velocity at the three observation points showed a high impact of the tidal wave producing ups and downs on DO concentrations, especially beside open boundaries with the Red Sea and reached variations of approximately 0.3 mgO_2_/l caused by the tide wave.

On the other hand, the effect of the tidal wave on the average water quality variables, except DO, was small. While points near boundaries showed a higher effect of tidal wave than the inner points of the lagoons, especially at points with high flow velocities’ fluctuation (i.e., point 1073 showed higher effect followed by point 2600 and point 1914).

#### Wind speeds

**Fig. 4:**
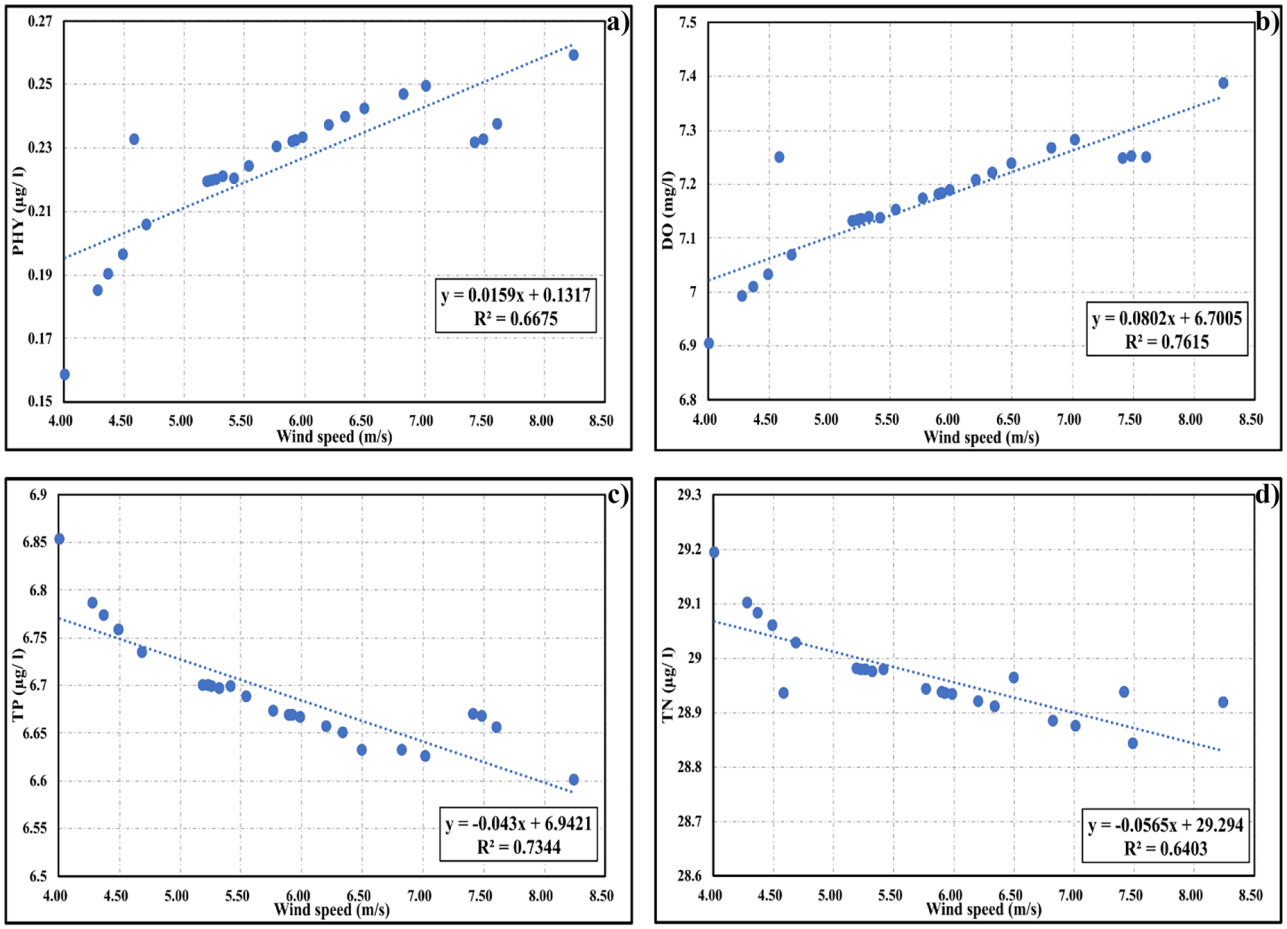
Relation between different wind speeds and water quality variables after 6 days of simulations: **a**) PHY (μg/l), **b**) DO (μg/l), **c**) TP (μg/l), **d**) TN (μg/l)

The effect of different wind speeds ranging from 8.24 to 4.01 m/s with the same wind direction of the northwest, which was computed during the study period of approximately 2 years was separately investigated. Firstly, the tidal wave was neglected to spotlight the effect of changes in wind speeds. Figure 4 clarifies the relationship between different wind speeds and water quality variables (PHY, DO, TP, TN), and the corresponding correlation coefficients *R*^2^ assuming linear regression. Further, water quality variables were simulated under the effect of the beforementioned different wind speeds considering the tide effect. Table 11 indicates the average difference percentage (RPD %) between the average water quality characteristics corresponding to the high wind speed (8.24 m/s) and the low wind speed (4.01 m/s) in the whole domain in both cases. Also, the lagoons were divided into three areas: upper area (area 1), bottom-left area (area 2), and bottom-right area (area 3) as shown in Fig. 1c. In this regard, the average difference in the most influenced area in the lagoons under the two cases of different mean wind neglecting and considering the tidal wave was also discussed.

**Table 11:** Average difference (%) of different water quality variables under the effect of decreasing wind speeds in the whole domain and the mostly affected area in the bottom left area (area 2) of the lagoons

	RPD (%)	PHY	PO_4_	POR	NO_3_	NOR	NH_4_	L	DO
Wind only	Whole domain	− 47.93	7.87	10.02	7.41	12.24	− 34.60	− 30.96	− 6.72
	Area 2	− 63.42	9.11	11.57	8.38	14.27	− 44.01	− 39.16	− 9.25
Tide and mean wind	Whole domain	− 22.89	1.17	0.03	1.28	1.12	− 14.59	− 13.75	− 4.39
	Area 2	− 26.86	1.50	0.02	1.53	1.33	− 17.25	− 16.29	− 4.14

It can be seen from Fig. 4 and Table 11 that changes in wind speeds are highly affecting the water quality variables in the lagoons producing correlation coefficients *R*^2^ ranging from 0.64 to 0.76. For instance, the average PHY and DO in the whole domain decreased by approximately 48% and 7%, respectively for decreasing wind speed. Nutrients such as PO_4_, POR, NO_3_, and NOR increased with decreasing of wind speeds by a rate ranging approximately from 7 to 13%. Area 2 was the most affected area by declining wind speeds with a reduction of 63% for PHY and 9% for DO.

Further, while considering the tidal wave, the effect of variations in wind speeds on different water quality variables decreased. For instance, the whole average PHY and DO decreased to approximately 23% and 4%, respectively influenced by decreasing wind speeds. Nutrients, on the other hand, showed increased values with smaller differences, when compared to the previous case with no tide wave. The bottom right part of the lagoons showed a relatively high influence with a difference of 27% for PHY and 4% for DO.

#### Wind speeds and wind directions

**Fig. 5:**
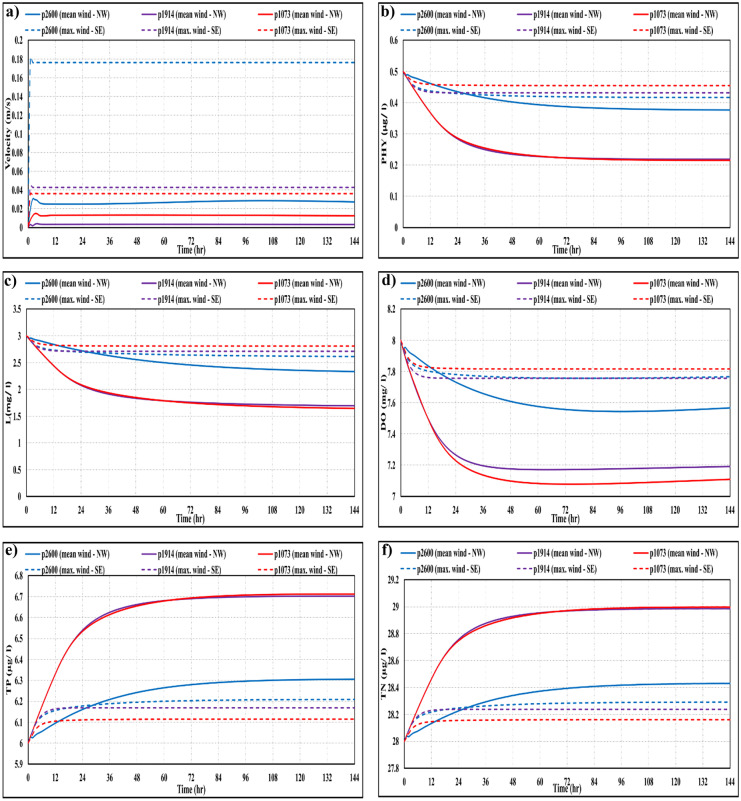
Temporal variation over 6 days considering the effect of mean wind from northwest direction and maximum wind from southeast direction at three observation points for **a**) flow velocity (m/s), **b**) PHY (μg/ l), **c**) L (mg/l), **d**) DO(mg/l), **e**) TP (μg/l), and **f**) TN (μg/l)

Water quality in the lagoons was discussed, herein, considering variations in both wind speeds and wind directions ignoring the tide effect. From metrological data in the study period (from 2015 to the end of 2017), the mean wind was 5.24 m/s from the northwest direction, while the maximum wind was 20.8 m/s from the southeast direction. Different wind directions can play a chief role in the distribution of water quality variables close to inflow and outflow boundaries. Figure 5 shows different water quality variables at the three observation points 2600, 1073, and 1914 close to inflow/outflow boundaries (indicated in Fig. 1c) and the average values in both beforementioned scenarios and the corresponding flow velocities.

It can be seen from Fig. 5 that the water quality variables in both scenarios displayed no ups and downs in their variations as occurred in the case of tide effect only. Also, the maximum wind showed a relatively higher quality of water when compared to mean wind regardless of the wind direction and flow direction. For instance, lower nutrients and higher PHY and DO were observed in the case of maximum wind, when compared to mean wind. Also, the water quality variables in the three observation points in both scenarios were affected by flow direction (inflow or outflow point) and flow velocity. For instance, in the case of mean wind only, inflow point 2600 had a relatively higher water quality with high average DO concentration and low nutrients when compared to the outflow points 1073 and 1914. On the other hand, in the case of maximum wind only, outflow point 2600 had approximately close water quality to the water quality beside inflow points 1073 and 1914 owing to high outflow velocity.

#### Comparison between different weather conditions

**Fig. 6:**
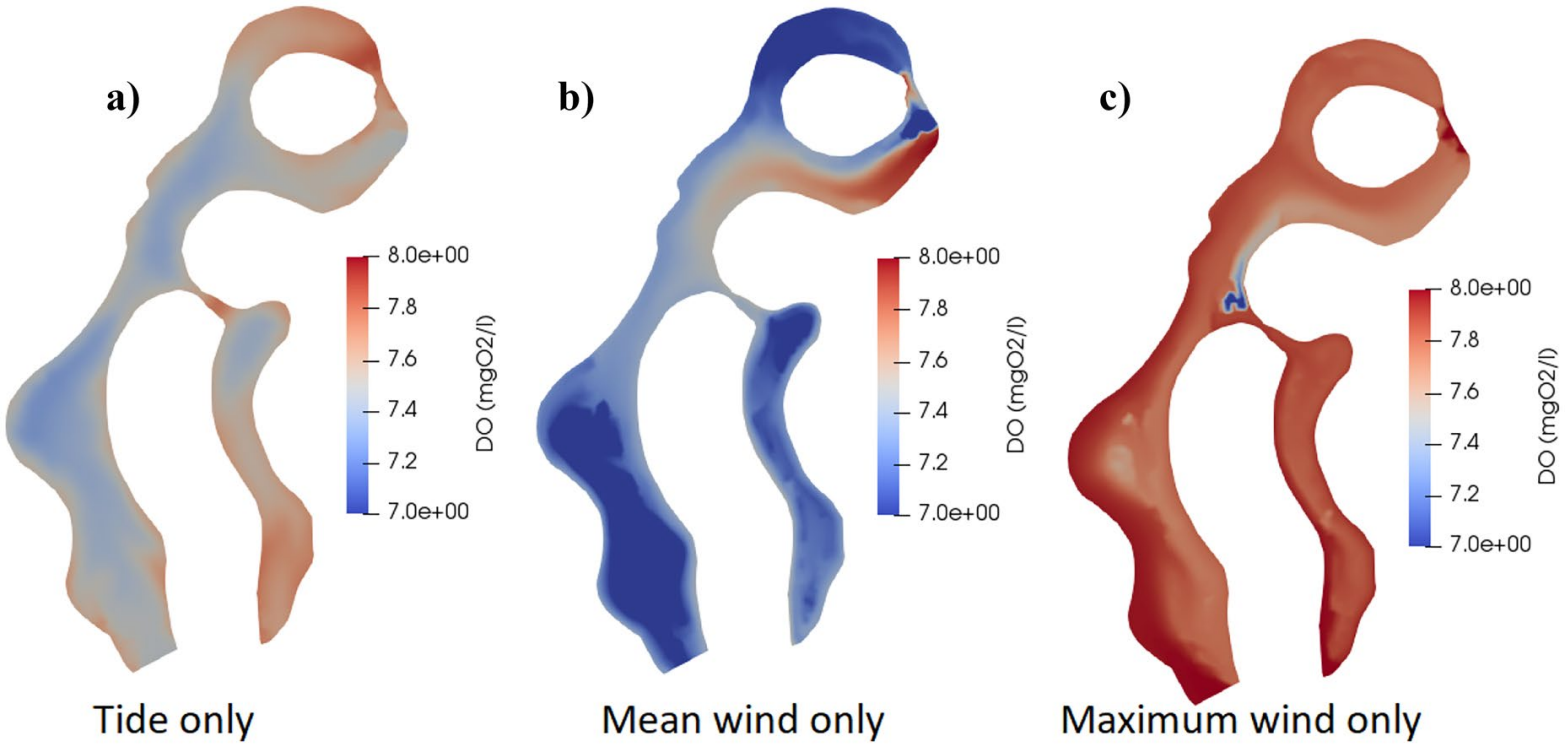
DO concentration (mg/l) in the whole domain after 6 days of simulation considering **a**) tide only, **b**) mean wind only, and **c**) maximum wind only

**Fig. 7:**
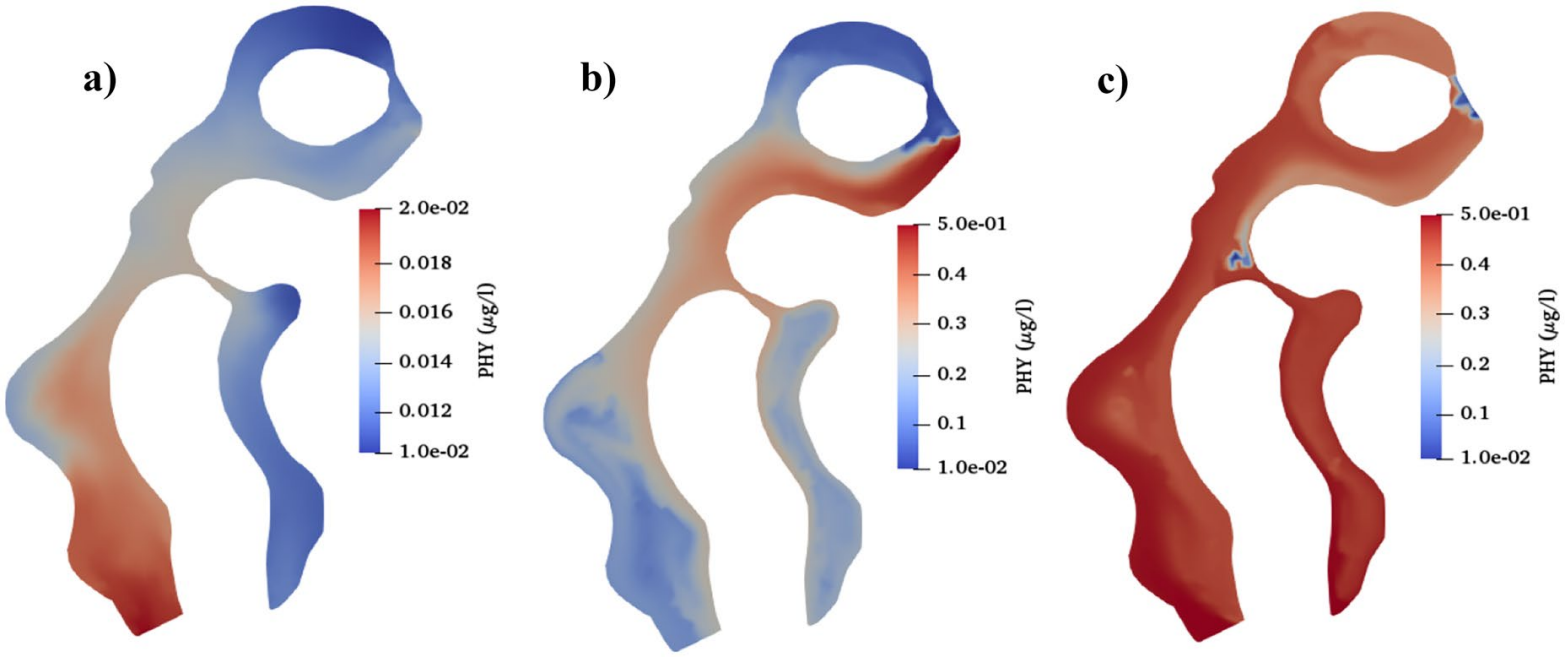
PHY concentration (μg/l) in the whole domain after 6 days of simulation considering **a**) tide only, **b**) mean wind only, and **c**) maximum wind only

Finally, Figs. 6 and 7 focus on the spatial PHY and DO at the end of 6 days of simulation under the effect of the three detailed weather conditions: tide only, mean wind only from the northwest direction and maximum wind only from the southeast direction. It can be concluded that each weather condition has a high effect on the spatial and temporal water quality in the lagoons.

### Brine discharge effects

#### Sensitivity analysis of different initial values of brine tracers’ concentrations and different inflow brine discharges

The effects of different initial values of brine tracers’ concentrations and different inflow brine discharges were discussed due to the lack of field data about the brine’s inflow. It is known that brine water has a relatively low water quality. Herein, water quality of brine effluent was expressed using different percental decreasing rates of water quality of the lagoons. For instance, a 10% decreasing rate means that the brine inflow has values of 90% for DO and 110% for nutrients and phosphorus from the initial values in the lagoons. The average water quality variables in the whole domain affected by the brine disposals and their corresponding values at observation point 845, which is located just beside the desalination boundary (Fig. 1c), are illustrated in Table 12. It is indicated from the table that the average water quality variables in the lagoons were not highly affected by different brine tracers’ concentrations or different inflow brine discharges. For instance, the average PHY presented neglected changes. DO reduced to an average value of 7.16 mg/l when the brine water is highly polluted (50% decreasing rate) and inflow has a high rate (1 m^3^/s), when compared to an average DO concentration of 7.62 mg/l at low brine pollution (10% decreasing rate) with low inflow rate (0.05 m^3^/s).

**Table 12:** Simulation scenarios, average water quality variables and water quality at point 845 beside the desalination boundary considering different tracers’ concentrations and different inflow brine discharges

Simulation scenarios		Average values				Point 845			
		PHY (μg/l)	TP (μg/l)	TN (μg/l)	DO (mg/l)	PHY (μg/l)	TP (μg/l)	TN (μg/l)	DO (mg/l)
Brine discharge of 0.1 m^3^/s with different decreasing rate of brine water characteristics	10%	0.13	6.97	29.48	7.62	0.22	6.85	29.86	7.48
	50%	0.12	7.31	31.13	7.48	0.16	7.28	38.44	6.46
Different brine discharges with 10% decreasing rate of brine water characteristics	0.05 m^3^/s	0.13	6.97	29.48	7.62	0.22	6.85	29.85	7.48
	0.2 m^3^/s	0.13	6.97	29.50	7.62	0.24	6.83	29.95	7.47
Different brine discharges with 50% decreasing rate of brine water characteristics	0.1 m^3^/s	0.12	7.31	31.13	7.48	0.16	8.68	38.44	6.46
	0.5 m^3^/s	0.12	7.51	32.19	7.38	0.22	10.74	49.32	5.39
	1 m^3^/s	0.13	8.00	34.74	7.16	0.25	11.85	55.23	4.95

On the other hand, point 845 was highly sensitive to changes in tracers’ concentrations and brine inflow owing to its location near the desalination inflow. For instance, the difference in the degree of pollution in brine discharge (10% and 50% decreasing ratios of the initial water quality variables) decreased PHY from 0.22 to 0.16 μg/l and DO from 7.48 to 6.46 mg/l, while TN and TP increased from 6.85 to 7.28 μg/l and from 29.86 to 38.44 μg/l, respectively. Different inflow brine discharges did not show high effects in case of low water pollution (10% decreasing rate) and were small compared to inflow brines range from 0.05 to 0.2 m^3^/s. On the other hand, increasing the brine inflow rate with highly polluted water (50% decreasing rate) could highly decrease water quality beside the desalination plant. For instance, DO decreased from 6.46 to 4.95 mg/l owing to the increase of inflow brine from 0.1 to 1 m^3^/s.

#### Different weather conditions

**Fig. 8:**
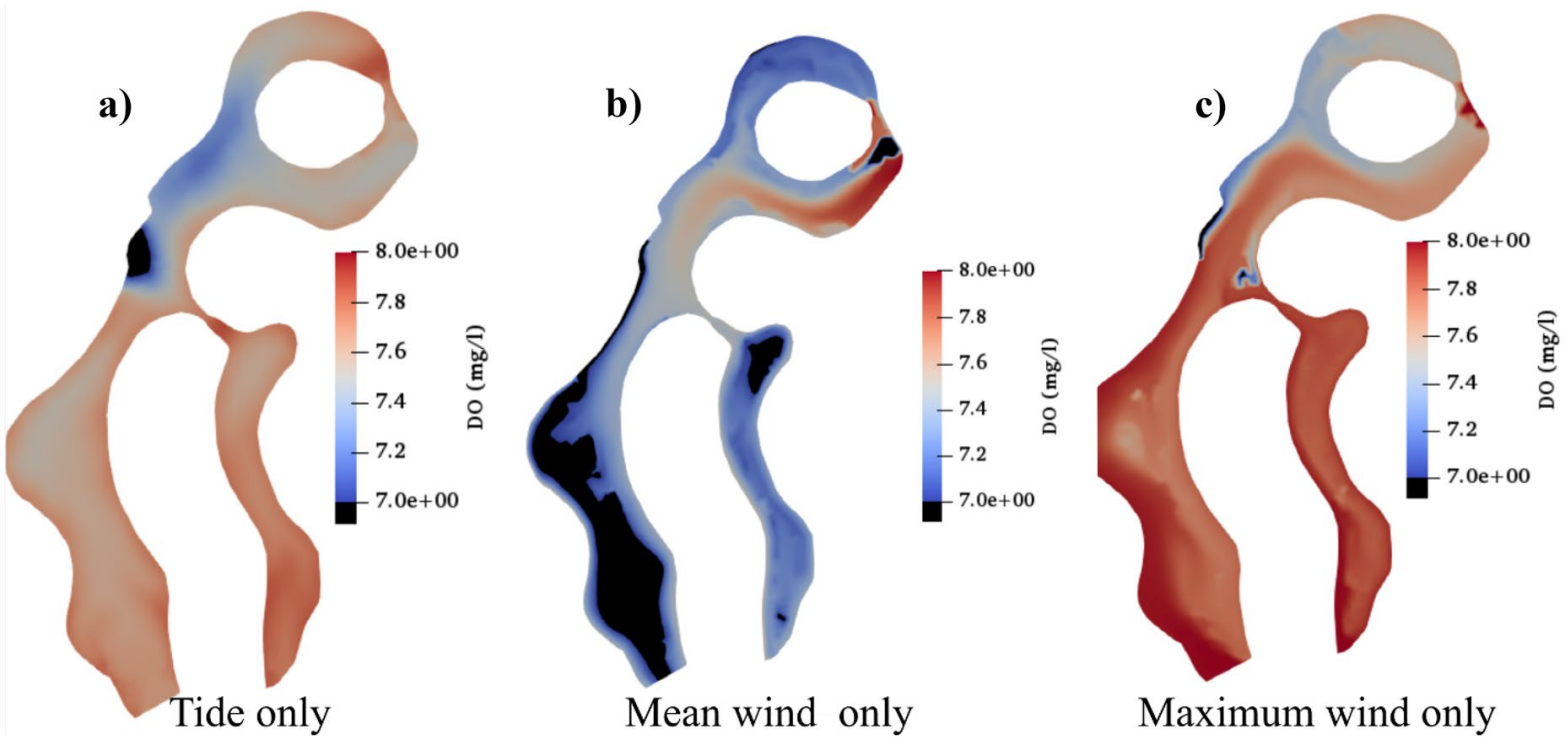
DO concentration (mg/l) in the whole domain after 8 days of simulation affected by the nearby desalination plant considering **a**) tide only, **b**) mean wind only, and **c**) maximum wind only

Finally, the propagation of brine disposals in the lagoons was discussed under the effect of three weather conditions: tide only, mean wind only, and high wind only. The same inflow of 0.1 m^3^/s with tracers’ concentration with a 20% decreasing rate was used for the three scenarios. Figure 8 displays the effect of brine disposals on DO as a main indicator of water quality in the lagoons and its propagation. The brine movement was significantly influenced by tide and wind conditions. It mainly concentrated beside the desalination boundary and spread upward with a low rate to leave the lagoons through the top boundary under the effect of the tidal wave, whilst it followed mainly the same direction of the mean wind to outflow through the left bottom boundary in the second scenario. Finally, the maximum wind direction once again drove the brine upwardly and helped it to leave the domain rapidly.

### Precautions for preservation of water quality in the lagoons

The water quality in the lagoons has been investigated under the effect of different precautions or changes in the hydraulics and the operating system of the nearby desalination plant aiming for preservation of the water quality in the lagoons. From the simulations, new recommendations for the decision-makers are presented and can help in the preservation of the water quality in the lagoons. For instance, larger inflow boundaries help to increase DO from 7.48 to 7.76 mg/l, as shown in Table 13. Also, increasing the DO concentration of inflow water from 7 to 8 mg/l helps to increase the average DO concentration in the whole domain from 6.66 to 7.54 mg/l in only 4 days. Moreover, the operation of the desalination plant plays a vital role in the disposal propagation in the domain. Consequently, it is recommended to inject the brine in time of tide with mean wind and not in time of tide only to quickly get rid of disposals and seeking better water quality, as indicated in Table 13.

**Table 13:** Precaution scenarios and their effect on the average water quality in the lagoons

Simulation scenarios	Average values							
	PHY (μg/l)	PO_4_ (μg/l)	POR (μg/l)	NO_3_ (μg/l)	NOR (μg/l)	NH_4_ (μg/l)	L (mg/l)	DO (mg/l)
Original inflow boundaries	0.19	2.30	4.47	17.82	6.46	4.79	1.57	7.48
Larger inflow boundaries	0.36	2.58	3.76	15.60	5.17	7.71	2.42	7.76
Brine injection under effect of tide wave	0.20	5.97	4.80	20.31	6.89	5.99	1.92	7.00
Brine injection under effect of mean wind only	0.27	2.33	3.81	15.55	5.35	6.13	1.94	6.95
Brine injection under effect of tide and mean wind	0.20	2.63	4.64	19.13	6.66	5.86	1.88	7.34

## Discussion

This work presents a complete insight of the water quality of artificial lagoons in El Gouna City. To the authors’ knowledge, no previous studies discussed the water quality problems in manmade lagoons. Moreover, the advanced TELEMAC-EUTRO-WAQTEL module was, for the first time, applied to investigate the water characteristics in the lagoons using more than 25 input parameters. In this regard, a sensitivity analysis of different input parameters in the newly applied module was firstly conducted. The eutrophication problem in the lagoons displayed high sensitivity to changes in many of the input parameters. For instance, both the maximum growth rate of phytoplankton (*C*_max_) and the phosphate half-saturation constant (KP) highly affected PHY, PO_4_, NOR, and POR. Also, the nitrification rate at 20 °C (*K*_520_) had an obvious effect on both NO_3_ and NH_4_. L, and DO, on the other hand, depended mostly on the degradation load of L and benthic demand, respectively.

Afterwards, the effect of different meteorological data such as water temperature, tide wave, and wind speed on different water quality parameters was investigated. In a globally warming climate, air temperature is expected to increase by 1.1–6.4 °C by 2100, which, in turn, rapidly increases water temperature (Anthony et al., 2009). Moreover, Langodan et al. (2017) revealed the future definite tendency toward lowering the strength of wind speed in the Red Sea. Additionally, this work sheds new light on the effect of different wind directions on the water characteristics. The results showed that the high water temperature showed a negative obvious effect on some of the water quality characteristics as the increase of water temperature decreased DO and increased nutrients pollutants such as NO_3_, NOR, and PO_4_. The obtained negative effects of water temperature on different water parameters concur with previous studies by McLusky and Elliott (2007), Park et al. (2013), Post et al. (2018), Trombetta et al. (2019). Moreover, for the tide effect scenario, the fluctuations of water depth in case of tide wave also created a wave of ups and downs in water quality of points near boundaries, especially DO. Concentrations of DO fluctuated with approximately 0.3 mgO_2_/l differences between ups and downs corresponding to 0.6 m amplitude of tide wave. The obtained results concur with previous study by Zhu et al. (2017), who found regular and periodic variations in DO concentrations following the tidal cycles in the study area. Also, other pollutants showed ups and downs fluctuations near the boundaries affected mostly by high fluctuation in flow velocities. On the other hand, the average values of the water pollutants showed minor fluctuations of ups and downs inside the lagoons and away from boundaries.

The applied wind speed and direction had a high effect on the eutrophication problem in the lagoons. For instance, considering the effect of different wind speeds, decreasing wind speed from the same wind direction was negatively affecting water quality variables in the lagoons, especially on the bottom left side. The high negative effect of decreasing wind speeds decreased when tide wave was combined with the effect of the differences in wind speeds. The negative effect of the expected decrease in wind speeds on the water quality in the lagoons concurs with a previous study by Deng et al. (2018), who found that decreasing wind speed coincided with increased P in their studied shallow eutrophic lake. On the other hand, the wind direction also had a high impact on the spatial water quality variables at inflow and outflow boundaries. For instance, points beside inflow boundaries had relatively higher PHY and DO values and lower nutrients, when compared to outflow boundaries, especially at low wind speeds.

El Gouna lagoons receive binary discharge from a nearby desalination plant, which alter the water characteristics in the lagoons. To date, the effect of desalination effluents on water characteristics in coastal areas has not been comprehensively investigated yet, despite the research interest on desalinated water characteristics is raising. In this regard, this work focuses on the harmful effect of pollution from the nearby desalination plant on the eutrophication process in the lagoons considering different weather conditions and different pollution intensity of desalination’s effluent. Sensitivity analysis of different discharges and pollutions from the nearby desalination plant showed that different initial tracer concentrations did not highly affect the average water quality in the lagoons: however, the area just beside the inlet of the binary discharge showed a high negative effect, especially in the case of a highly polluted brine with a large inflow rate. Moreover, the simulation showed that the propagation of the tracers from desalination outflow was strongly affected by the weather conditions, as they moved upward in case of tide wave and maximum wind scenario, while they moved downward in case of the mean wind.

Finally, this study serves as a steppingstone for further work toward new precautions or changes in the hydraulics and the operation system of nearby desalinations plant seeking better water quality. For instance, larger inflow boundaries and injecting tracer in time of tide and mean wind instead of in the time of tide only or mean wind only are new suggested precautions.

## Conclusions

A two-dimensional vertically averaged water quality numerical model using the TELEMAC-EUTRO-WAQTEL module has been applied to investigate the eutrophication water pollution problem in artificial lagoons of El Gouna City, which are highly stressed by the increasing tourism activities. Eight water quality variables were discussed: PHY, DO, PO_4_, POR, NO_3_, NOR, NH_4_, and L. The lagoons were found highly sensitive to metrological parameters such as water temperature, tide cycles, and wind patterns. The negative effect of the upcoming climate changes of higher water temperature and low wind speeds on the water quality in the lagoons was discussed. Moreover, the lagoons were also investigated under the negative effect from the nearby desalination plant, which throws its binary discharge in the lagoons. Finally, new precautions in the hydraulics and the operation system of nearby desalinations plant were discussed. Such precautions and a reduction in the external nutrient loading are needed, and their importance will increase in a future warmer world to help the lagoons facing the future climate circumstances and the polluted effluent from desalination plant.

This investigation is regarded as a preliminary and crucial step toward gaining a comprehensive understanding of water quality in El Gouna artificial lagoons and their environmental preservation. Having a broader picture of the water quality in the lagoons is critical for initiating dialogs with stakeholders and decision-makers in the area, as well as embracing mitigation steps to safeguard El Gouna’s ecosystem and the adjacent coral reefs.

## Data Availability

All data generated or analyzed during this study are included in this published article.

## References

[CR1] Abouelsaad O, Matta E, Hinkelmann R (2022). Hydrodynamic response of artificial lagoons considering tide, wind and tracer—Case study El Gouna, Egypt. Regional Studies in Marine Science.

[CR2] Abouelsaad, O., Matta, E., Omar, M. E. M., & Hinkelmann, R. (2022b). Numerical simulation of dissolved oxygen as a water quality indicator in artificial lagoons–Case study El Gouna, Egypt. *Regional Studies in Marine Science*, 102697.

[CR3] Al-Jabari, M. (2018). Numerical simulation of exchange processes between lagoons and the Red Sea in El Gouna, Egypt. Master Thesis, Water Engineering department, Technische Universität Berlin, Germany.

[CR4] Anthony, A., Atwood, J., August, P., Byron, C., Cobb, S., Foster, C., Fry, C., Gold, A., Hagos, K., & Heffner, L. (2009). Coastal lagoons and climate change: ecological and social ramifications in US Atlantic and Gulf coast ecosystems. *Ecology and Society*, *14*.

[CR5] Chao X, Jia Y, Shields FD, Wang SSY, Cooper CM (2010). Three-dimensional numerical simulation of water quality and sediment-associated processes with application to a Mississippi Delta lake. Journal of Environmental Management.

[CR6] Churchill, M. A., Elmore, H. L., & Buckingham, R. A. (1964). The prediction of stream reaeration rates, in: advances in water pollution research, proceedings of the international conference held in london. Pergamon Press LTD, pp. 89–136. 10.1016/b978-1-4832-8391-3.50015-4

[CR7] De Jonge, V. N., Elliott, M., & Orive, E. (2002). Causes, historical development, effects and future challenges of a common environmental problem: eutrophication. In: *Nutrients and Eutrophication in Estuaries and Coastal Waters*. Springer, pp. 1–19.

[CR8] de la Presa Owens S, Innis SM (1999). Docosahexaenoic and arachidonic acid prevent a decrease in dopaminergic and serotoninergic neurotransmitters in frontal cortex caused by a linoleic and α-linolenic acid deficient diet in formula-fed piglets. The Journal of Nutrition.

[CR9] Deng J, Paerl HW, Qin B, Zhang Y, Zhu G, Jeppesen E, Cai Y, Xu H (2018). Climatically-modulated decline in wind speed may strongly affect eutrophication in shallow lakes. Science of the Total Environment.

[CR10] Eichelberger S, McCaa J, Nijssen B, Wood A (2008). Climate change effects on wind speed. North American Windpower.

[CR11] Fahmy MA, Fattah LMA, Abdel-Halim AM, Aly-Eldeen MA, Abo-El-Khair EM, Ahdy HH, Ahdy HH, Hemeilly A, El-Soud AA, Shreadah MA (2016). Evaluation of the quality for the Egyptian Red Sea coastal waters during 2011–2013. Journal of Environmental Protection.

[CR12] Fatema K, Omar W, Isa M (2016). Effects of tidal events on the water quality in the Merbok Estuary, Kedah, Malaysia. Journal of Environmental Science and Natural Resources.

[CR13] Fernández B, García A, García J, Álvarez C, Antonio J, Cortezón R (2012). A model for describing the eutrophication in a heavily regulated coastal lagoon. Application to the Albufera of Valencia ( Spain ). Journal of Environmental Management.

[CR14] Flather, R. A. (1976). Results from a storm surge prediction model of the North European Contenental Shelf for April, November and December 1973. Technical Report 24, Wormley, UK, Institute of Oceanographic Sciences, 37 pp.

[CR15] Gasim MB, Khalid NA, Muhamad H (2015). The influence of tidal activities on water quality of paka river Terengganu, Malaysia. Malaysian Journal of Analytical Sciences.

[CR16] George G, Hurley M, Hewitt D (2007). The impact of climate change on the physical characteristics of the larger lakes in the English Lake District. Freshwater Biology.

[CR17] Guildford SJ, Hecky RE (2000). Total nitrogen, total phosphorus, and nutrient limitation in lakes and oceans: Is there a common relationship?. Limnology and Oceanography.

[CR18] Haider H, Ali W, Haydar S (2013). Evaluation of various relationships of reaeration rate coefficient for modeling dissolved oxygen in a river with extreme flow variations in Pakistan. Hydrological Processes.

[CR19] Hervouet, J. M., & Ata, R. (2017). Technical report: User manual of opensource software TELEMAC-2D V7P2, Report. EDF-R&D. www.opentelemac.org

[CR20] Kay, R., & Alder, J. (2005). *Coastal planning and management* (2nd ed.). CRC Press, London, England: Taylor & Francis Group. 10.1201/9781315272634

[CR21] Kim JS, Seo IW, Lyu S, Kwak S (2018). Modeling water temperature effect in diatom (Stephanodiscus hantzschii) prediction in eutrophic rivers using a 2D contaminant transport model. Journal of Hydro-Environment Research.

[CR22] Kroon FJ, Thorburn P, Schaffelke B, Whitten S (2016). Towards protecting the Great Barrier Reef from land-based pollution. Global Change Biology.

[CR23] Kunarso, Zainuri M, Ario R, Munandar B, Prayogi H (2018). Impact of monsoon to aquatic productivity and fish landing at Pesawaran Regency Waters. IOP Conference Series: Earth and Environmental Science.

[CR24] Langodan S, Cavaleri L, Vishwanadhapalli Y, Pomaro A, Bertotti L, Hoteit I (2017). The climatology of the Red Sea–part 1: The wind. International Journal of Climatology.

[CR25] Liu X, Feng J, Wang Y (2019). Chlorophyll a predictability and relative importance of factors governing lake phytoplankton at different timescales. Science of the Total Environment.

[CR26] Liu Y, Guo H, Wang L, Dai Y, Zhang X, Li Z, He B (2006). Dynamic phosphorus budget for lake-watershed ecosystems. Journal of Environmental Sciences.

[CR27] Marlina N, Melyta D (2019). Analysis effect of cloud cover, wind speed, and water temperature to BOD and DO concentration using QUAL2Kw model (case study in Winongo River, Yogyakarta). MATEC Web of Conferences.

[CR28] McLusky DS, Elliott M (2007). Transitional waters: A new approach, semantics or just muddying the waters?. Estuarine, Coastal and Shelf Science.

[CR29] Ménesguen A, Lacroix G (2018). Science of the total environment modelling the marine eutrophication: A review. Science of the Total Environment.

[CR30] Morgan EJ, Lavric JV, Arévalo-Martínez DL, Bange HW, Steinhoff T, Seifert T, Heimann M (2019). Air-sea fluxes of greenhouse gases and oxygen in the northern Benguela Current region during upwelling events. Biogeosciences.

[CR31] Nazari-Sharabian, M., Ahmad, S., & Moses, K. (2018). Climate change and groundwater: A short review Climate change and groundwater: A short review. *Engineering, Technology and Applied Science Research*, 8, No.

[CR32] Nixon SW, Granger S, Buckley BA, Lamont M, Rowell B (2004). A one hundred and seventeen year coastal water temperature record from Woods Hole, Massachusetts. Estuaries.

[CR33] O’Connor DJ, Dobbins WE (1958). Mechanism of reaeration in natural streams. Transactions of the American Society of Civil Engineers.

[CR34] Örnólfsdóttir EB, Lumsden SE, Pinckney JL (2004). Phytoplankton community growth-rate response to nutrient pulses in a shallow turbid estuary, Galveston Bay, Texas. Journal of Plankton Research.

[CR35] Pal, N. (2020). Eutrophication- An Ecological Men- ace 559–561.

[CR36] Park JY, Park GA, Kim SJ (2013). Assessment of future climate change impact on water quality of chungju lake, South Korea, Using WASP Coupled with SWAT. Journal of the American Water Resources Association.

[CR37] Park RA (1980). Handbook of environmental data and ecological parameters. Endeavour.

[CR38] Post CJ, Cope MP, Gerard PD, Masto NM, Vine JR, Stiglitz RY, Hallstrom JO, Newman JC, Mikhailova EA (2018). Monitoring spatial and temporal variation of dissolved oxygen and water temperature in the Savannah River using a sensor network. Environmental Monitoring and Assessment.

[CR39] Purnaini R, Sudarmadji, Purwono S (2018). Tidal influence on water quality of Kapuas Kecil River Downstream. E3S Web of Conferences.

[CR40] Richardson, J. L., Arndt, J. L., & Montgomery, J. A. (2001). Hydrology of wetland and related soils. Wetland soils–Genesis, hydrology, landscapes, and classification 35–84.

[CR41] Rigosi A, Marcé R, Escot C, Rueda FJ (2011). A calibration strategy for dynamic succession models including several phytoplankton groups. Environmental Modelling & Software.

[CR42] Sepulveda-Jauregui A, Hoyos-Santillan J, Martinez-Cruz K, Anthony KMW, Casper P, Belmonte-Izquierdo Y, Thalasso F (2018). Eutrophication exacerbates the impact of climate warming on lake methane emission. Science of the Total Environment.

[CR43] Shimoda Y, Arhonditsis GB (2016). Phytoplankton functional type modelling: Running before we can walk? A critical evaluation of the current state of knowledge. Ecological Modelling.

[CR44] Smetacek V, Zingone A (2013). Green and golden seaweed tides on the rise. Nature.

[CR45] Tchobanoglous G, Schroeder EE (1985). Water quality: Characteristics, modeling, modification.

[CR46] Trombetta T, Vidussi F, Mas S, Parin D, Simier M, Mostajir B (2019). Water temperature drives phytoplankton blooms in coastal waters. PLoS ONE.

[CR47] Wang X, Zhang S, Liu S, Chen J (2012). A two-dimensional numerical model for eutrophication in Baiyangdian Lake. Frontiers of Environmental Science and Engineering in China.

[CR48] Wirasatriya, A., Kunarso, K., Maslukah, L., Satriadi, A., & Armanto, R. D. (2018). Different responses of chlorophyll-a concentration and Sea Surface Temperature (SST) on southeasterly wind blowing in the Sunda Strait. *IOP Conference Series: Earth and Environmental Science*, *139*. 10.1088/1755-1315/139/1/012028

[CR49] Wool, T. A., Ambrose, R. B., Martin, J. L., Comer, E. A., & Tech, T. (2006). Water quality analysis simulation program (WASP). User’s manual, Version 6. Washington, DC: US Environmental Protection Agency.

[CR50] Woznicki SA, Nejadhashemi AP, Tang Y, Wang L (2016). Large-scale climate change vulnerability assessment of stream health. Ecological Indicators.

[CR51] Wu T, Luo L, Qin B, Cui G, Yu Z, Yao Z (2009). A vertically integrated eutrophication model and its application to a river-style reservoir - Fuchunjiang, China. Journal of Environmental Sciences.

[CR52] Wurtsbaugh, W. A. (2019). Nutrients , eutrophication and harmful algal blooms along the freshwater to marine continuum. 1–27. 10.1002/wat2.1373

[CR53] Wurtsbaugh WA, Paerl HW, Dodds WK (2019). Nutrients, eutrophication and harmful algal blooms along the freshwater to marine continuum. Wires Water.

[CR54] Xia R, Zhang Y, Critto A, Wu J, Fan J, Zheng Z, Zhang Y (2016). The potential impacts of climate change factors on freshwater eutrophication: Implications for research and countermeasures of water management in China. Sustainability.

[CR55] Yang XE, Wu X, Hao HL, He ZL (2008). Mechanisms and assessment of water eutrophication. Journal of Zhejiang University: Science B.

[CR56] Zhang, W., Xu, Q., Wang, X., Hu, X., Wang, C., Pang, Y., Hu, Y., Zhao, Y., & Zhao, X. (2017). Spatiotemporal distribution of eutrophication in Lake Tai as affected by wind. *Water (Switzerland)*, *9*. 10.3390/w9030200

[CR57] Zhu ZY, Wu H, Liu SM, Wu Y, Huang DJ, Zhang J, Zhang GS (2017). Hypoxia off the Changjiang (Yangtze River) estuary and in the adjacent East China Sea: Quantitative approaches to estimating the tidal impact and nutrient regeneration. Marine Pollution Bulletin.

